# Supplementary material for: Development of Novel Inhibitors Targeting the D-Box of the DNA Binding Domain of Androgen Receptor
Source: Int J Mol Sci. 2021 Mar 2;22(5):2493. doi: 10.3390/ijms22052493 (PMC7958344; doi:10.3390/ijms22052493)

Supplementary material

Supplementary Table 1. List of top actives. Hit compounds VPC-17160 and VPC-17281 are in bold.

Compound ID VPC-*	Inhibition Dox-V7 (10 µM)	eGFP FL (12 µM)	Nanoluc Counterscreen (10 µM)	IC_{50} Dox-V7 (µM)	IC_{50} eGFP AR-FL (µM)
**17005**				**10**	**0.8**
17116	10	111	50-60%		25
17121	10	73	10-20%		15
17156	100	44	50%		25
17159	42	56	10%		10
**17160**	**70**	**76**	**20%**	**6**	**2**
17166	90		60%		
17177	80	43	>75%		
17178	80	28	>75%		
17179	80	17	>75%		
17181	90	43	>75%		
17184	80	73			>25
17199	50	95			10
17226	45	76	25%		5
17246	60	66	30%		>10
17247	45	102			8
17248	90	125	50%	3	3
17261	10	102	15%		10
**17281**	**70**	**100**	**10%**	**6**	**5**
17303	50	100			10
17336	50	100			6

Supplementary Figure 1. Pharmacophore model based on VPC-17005. The protein surface is shown in black with blue indicating positively charged areas and red indicating negatively charged areas. The ligand is shown in purple with the aromatic pharmacophore feature in orange and the hydrogen bond acceptor feature in cyan.

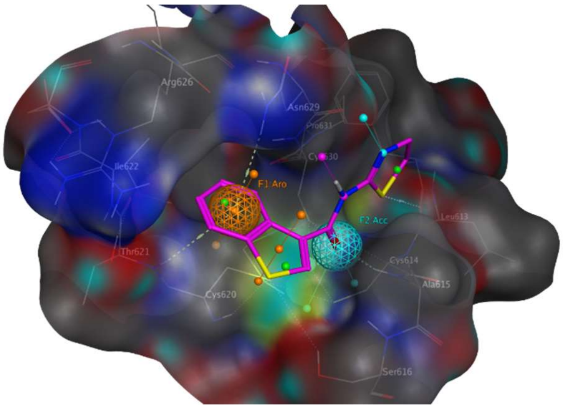

Supplement: Supplementary file 1 [file ijms-22-02493-s001.pdf]
